# Interactive visualization of multi-data-set Rietveld analyses using *Cinema:Debye-Scherrer*

**DOI:** 10.1107/S1600576718003989

**Published:** 2018-05-09

**Authors:** Sven C. Vogel, Chris M. Biwer, David H. Rogers, James P. Ahrens, Robert E. Hackenberg, Drew Onken, Jianzhong Zhang

**Affiliations:** a Materials Science and Technology Division, Los Alamos National Laboratory, Los Alamos, NM 87545, USA; b Computing, Computational and Statistical Sciences Division, Los Alamos National Laboratory, Los Alamos, NM 87545, USA; c Sigma Manufacturing Sciences Division, Los Alamos National Laboratory, Los Alamos, NM 87545, USA; d Department of Physics, Wake Forest University, Winston-Salem, NC 27109, USA

**Keywords:** visualization, diffraction data analysis, automated analysis

## Abstract

A tool named *Cinema:Debye-Scherrer* to visualize the results of a series of Rietveld analyses is presented. The multi-axis visualization of the high-dimensional data sets resulting from powder diffraction analyses allows identification of analysis problems, prediction of suitable starting values, identification of gaps in the experimental parameter space and acceleration of scientific insight from the experimental data. The tool is demonstrated with analysis results from 59 U–Nb alloy samples with different compositions, annealing times and annealing temperatures as well as with a high-temperature study of the crystal structure of CsPbBr_3_. A script to extract parameters from a series of Rietveld analyses employing the widely used *GSAS* Rietveld software is also described. Both software tools are available for download.

## Abstract 2

A tool to visualize the results of a series of Rietveld analyses is presented, allowing identification of analysis problems, prediction of suitable starting values and acceleration of scientific insight from the experimental data.

## 1. Introduction   

Modern diffraction instruments at neutron and synchrotron sources produce data sets at ever faster rates. Rietveld analysis (Rietveld, 1969 ▸; Young, 1993 ▸) is the standard analysis method for powder diffraction data, fitting in many cases tens of parameters to derive crystallographic parameters (*e.g.* lattice parameters, atomic positions, atomic displacement parameters), microstructural parameters (*e.g.* micro-stresses or particle sizes from peak broadening or texture from peak intensities) and instrumental parameters. While some techniques and tools exist to automate the analysis, *e.g.* *seqgsas* in *GSAS* (Larson & Von Dreele, 2004 ▸), batch mode for *MAUD* (Lutterotti *et al.*, 1999 ▸; Lutterotti, 2011 ▸), the sequential analysis in *GSAS-2 * (Toby & Von Dreele, 2013 ▸), and *SrRietveld* for both *GSAS* and *Fullprof* (Tian *et al.*, 2013 ▸), they typically rely on the ability to use a template refinement that will converge when the experimental data are changed to a new data set and the set of refinable parameters remains identical to the template. Script-based approaches such as gsaslanguage (Vogel, 2011 ▸) allow more flexibility by applying refinement strategies even when a template refinement is used for a new data set (*e.g.* fix all parameters but scale and background and refine, then refine parameters incrementally) by querying parameters to allow conditional refinements (*e.g.* refine lattice parameters only above a threshold for the volume fraction). While these tools automate the analysis, the inspection of the results (parameter values as well as graphical inspection of experimental intensities and fit), the prediction of parameter starting values likely to lead to convergence of the refinement and ultimately the scientific insight from a large data set of results from several tens or hundreds of Rietveld refinements still pose a challenge.

Since Rietveld refinements frequently vary tens or even hundreds of parameters, inspecting the refined parameters of a larger series of Rietveld refinements is not trivial, with simultaneous multi-histogram refinements complicating the situation. A single diverged parameter can invalidate the analysis of an entire experimental data set, making the identification of outliers mandatory. A tool allowing rapid identification of the parameter leading to divergence in some data sets is to the best of the authors’ knowledge unavailable at present. Similarly, a tool to efficiently inspect tens or hundreds of single- or multi-histogram Rietveld analyses graphically is missing. Addressing problems related to the experiment, for example too small or too large sample volumes, may be crucial for successful beam time if real-time analysis is available. In many cases, trial-and-error approaches or cumbersome single peak fitting are necessary to identify starting values for parameters (*e.g.* lattice parameters) of unknown structures (*e.g.* in a solid solution or at elevated temperature or pressure). A tool utilizing successful refinement results to predict starting values likely to lead to convergence on the basis of external parameters (*e.g.* composition or temperature) can accelerate the data analysis. Finally, a tool to identify trends in the highly multi-dimensional refinement results, correlating external parameters (sample composition or temperature) with refinement results (phase volume fractions, atomic positions), would accelerate the pace of scientific insight from a series of powder diffraction analyses. A tool that readily visualizes relationships between any two parameters would expedite analyses uncovering new scientific discoveries. If real-time data analysis is available at the beamline during the experiment, early identification of some relationships may direct the experimental design if the analyst notices a particular region of interest in the parameter space that they would like to sample more finely. In addition, an interactive graphic interface with a simple input format and connection to automated workflows, *i.e.* automated data collection and data analysis, would alleviate some of the user-interface challenges between workflow data and visualization tools.

While tools to visualize raw scattering data exist, *e.g.* *Mantid* (Arnold *et al.*, 2014 ▸), the tool described here specifically deals with visualization of analyzed scattering or diffraction data. Similarly, tools to visualize individual crystal structures exist, *e.g.* *VESTA* (Momma & Izumi, 2011 ▸), but results of parametric studies or diffraction parameters related to microstructural parameters such as peak width cannot be visualized with such tools. The commercial *PolySNAP3* visualization and analysis tool for high-throughput diffraction data (Barr *et al.*, 2009 ▸) provides statistical analysis of the raw diffraction data, but not Rietveld analysis results.

In this article, we introduce an interactive data exploration tool, *Cinema:Debye-Scherrer*, and demonstrate how it can aid in the validation of Rietveld analyses, scientific discovery and experimental design. In §2, we present the visualization tool *Cinema:Debye-Scherrer*, which allows users to quickly get an overview of large data sets in order to identify outliers and interesting relationships between parameters. A script to extract relevant data from *GSAS* Rietveld refinements is also introduced. In §3, we demonstrate our visualization approach with *Cinema:Debye-Scherrer* on example data sets from parametric diffraction studies of uranium–niobium samples and caesium plumbo tribromide. Finally, in §4 we summarize our approach to the visualization of diffraction data results.

## 2. Methods   

### 2.1. *Cinema:Debye-Scherrer*   

*Cinema* is an open-source image-based approach to extreme-scale data analysis predominantly developed at Los Alamos National Laboratory as part of the Exascale Computing Project. *Cinema* addresses the problem that in extreme-scale scientific simulations data analytics runs the risk of being a bottleneck to scientific discovery, by providing *in situ* visualization as an integral part of the workflow. *Cinema* provides a novel framework for a highly interactive, image-based approach to data analysis that promotes exploration of, for example, simulation results or in this particular case Rietveld analysis results. Custom-developed pipelines as interfaces for data stored in different formats and viewers for data display provide flexibility to adapt *Cinema* for different problems. The adaptation of the *Cinema* viewers to view a powder diffraction data set and the subset of viewers chosen for inspection of Rietveld analysis results was named *Cinema:Debye-Scherrer* in reference to the Debye–Scherrer camera.

#### 2.1.1. Input format   

The input specifications to *Cinema* were chosen with generality in mind. The file format is a comma-separated value (CSV) file where the final set of columns may contain paths to images. This is referred to as a *Cinema* database. The *Cinema* database specifications were designed for an image-based approach for the visualization of extreme-scale (>10^15^ FLOPS) *in situ* simulation data  (O’Leary *et al.*, 2016 ▸); however, CSV files are a popular file format so the input to *Cinema:Debye-Scherrer* should be portable to many other applications as well.

#### 2.1.2. Web-based visualization interface   

**Figure 1:**
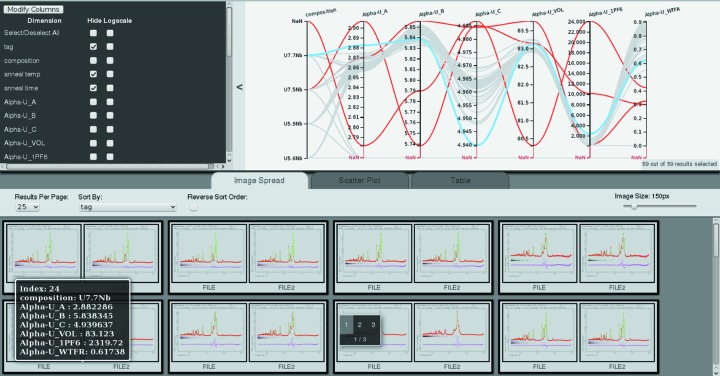
Screenshot of the *Cinema:Debye-Scherrer* web-based user interface.

*Cinema:Debye-Scherrer* is an interactive data exploration tool implemented with a web-based interface using the *Cinema Components* and *D3*  (Bostock *et al.*, 2011 ▸) JavaScript libraries. A web-based interface allows the visualizations to be displayed within web browsers for a versatile distribution across different platforms and the visualization code to be placed on a server for remote sharing between collaborators. Fig. 1 ▸ shows an overview of the *Cinema:Debye-Scherrer* interface which consists of several panels that display different types of visualizations or controls.

At the top of the interface is a parallel coordinates plot which shows a set of points in an *n*-dimensional space drawn across *n* vertical axes; there is one vertical axis for each dimension which may be numerical (*e.g.* lattice parameter value) or categorical (*e.g.* the composition shown on the first axis in the example). This visualization presents the analyst with an overview of an *n*-dimensional data set and the interactive interface allows the analyst to explore relationships between parameters. For example, anti-correlations and correlations are depicted as lines connecting axes that cross or do not cross, respectively. The vertical axes may be re-arranged to emphasize certain relationships in the data set. Interesting points or outliers may be highlighted (red lines in Fig. 1 ▸) and a subset of samples can be selected by dragging a box vertically across a particular axis. A panel to the side of the parallel coordinates plot contains display options such as hiding axes or changing a particular axis to a logarithmic scale.

At the bottom of the interface is a panel to switch between an image spread, scatter plot or sortable tabular display. The image spread can display multiple images for each sample in the data set. With the current interface to the *GSAS* Rietveld software, PNG files with measured data, Rietveld fit and difference curve are included for all histograms of a refinement. With slight modifications of the input file, photographs of samples, pole figures describing the texture or any other bitmaps could also be included. Scatter plots and tables are commonly used to inspect data sets from parametric diffraction studies, *e.g.* to plot unit-cell volume as a function of temperature or pressure. The scatter plot display in *Cinema:Debye-Scherrer* dynamically plots any two parameters from the input database. Examples of the scatter plot display are shown in Figs. 4 to 7.

Any dynamic selections or highlighted points from the parallel coordinates plots are communicated to the three other displays in the lower panel: selections of subsets by selecting regions on any axis (*e.g.* selecting ranges of lattice parameter values) will limit the displayed plots to the selected paths, marking of paths will highlight rows in the table view *etc*. Highlighting a certain image in the image spread will highlight the corresponding path (*e.g.* in Fig. 1 ▸ highlighting the left image of the two images corresponding to the second data set from the bottom right in the image spread, marked with the ‘1 2 3, 1/3’ indicator, results in highlighting of the path shown in blue in the multi-axis plot). Similarly, any highlighted selections from the scatter plot and tabular displays propagate to the other visualizations. Hovering over a particular point in any of the visualizations displays its information for the displayed database columns (*e.g.* bottom left of Fig. 1 ▸).

#### 2.1.3. Workflow integration   

A bash script to compile results from *GSAS* PVE files (files containing parameter name, value and estimated standard deviation) for all PVE files in a directory has been developed. The script uses the phase names defined in the *GSAS* experiment files (EXP) files and can accommodate phases being present only in a subset of EXP files as well as parameters only refined for certain files. A CSV file (readily produced with any spreadsheet software) with external parameters (*e.g.* sample mass, temperatures or annealing times) can be integrated. The result is a CSV file which can be processed by *Cinema:Debye-Scherrer*, spreadsheet programs or other plotting software. Bitmap files with the experimental data, fit and difference curve for all histograms included in an analysis are also created and referenced in the resulting file. The script, *gsas_prepare_cinema*, is part of the gsaslanguage package but also works without the entire package.

These lightweight tools are fast operations that do not add significant time to the analysis. The extraction of data from the 59 workflows described in this article is completed on a timescale of the order of seconds using *gsas_prepare_cinema*. In addition, the extracted data are quickly rendered with *Cinema:Debye-Scherrer*. For data sets of a thousand samples with tens of parameters, the results appear in the web browser in approximately 1 s.

#### 2.1.4. Distribution and documentation   

The source code and documentation of *Cinema:Debye-Scherrer* are available for download from https://github.com/cinemascience/cinema_debye_scherrer. New releases of *Cinema:Debye-Scherrer* will be posted at the same location. *Cinema:Debye-Scherrer* was built with *Cinema Components*, which is a JavaScript library to construct web-based visualization tools for *Cinema* databases. Some of the visualization capabilities in *Cinema:Debye-Scherrer* have been implemented in the *Cinema Components* library, which is available at https://github.com/cinemascience/cinema_components.

The script *gsas_prepare_cinema* which takes the output from the gsaslanguage workflows and converts it to a *Cinema* database is included in gsaslanguage which is available at https://github.com/Svennito/gsaslanguage.

## 3. Examples   

### 3.1. Uranium–niobium   

Fifty-nine samples with four different U–Nb compositions, annealing times from minutes to years, annealing temperatures from 300 to 600°C as well as varying sample sizes were characterized by neutron diffraction. Neutron powder diffraction data were collected on the HIPPO (High-Pressure Preferred Orientation) instrument (Wenk *et al.*, 2003 ▸) at the pulsed neutron spallation source LANSCE (Lisowski & Schoenberg, 2006 ▸) using a robotic sample changer (Losko *et al.*, 2014 ▸). Data sets for 0, 67.5 and 90° rotations around the vertical axis were collected, allowing texture analysis following procedures described by Wenk *et al.* (2010 ▸). The data collected from the 1200 ^3^He detector tubes of the HIPPO instrument, arranged on 45 detector panels on five rings around the incident beam, were integrated for the five rings, resulting in five histograms per run. The histogram data from the three rotations were then added for each ring, resulting in five histograms for each sample with random grain orientations as any weak to moderate texture is randomized by this procedure. All data sets were analyzed with the same gsaslanguage (Vogel, 2011 ▸) script. Consistent with the U–Nb phase diagram and applicable time–temperature–transformation diagrams (Jack­son, 1971 ▸; Hackenberg *et al.*, 2011 ▸, 2015 ▸, 2017 ▸), monoclinic α′′-U–Nb, tetragonal γ^0^-U–Nb, orthorhombic α-U and α′-U–Nb, and cubic γ-U were part of the analysis. In some cases urania UO_2_ (from sample oxidation) and aluminium (from sample holders) were detected. The phases to be included in the analysis were provided as command-line parameters to the analysis script. A second script executed the analysis of all data sets in batch mode. Adjusting the U–Nb phase composition *via* the site-occupation factors in the cubic γ phase on the basis of the Jackson equation (Jackson, 1970 ▸), relating lattice parameter to composition, increased the accuracy of weight fractions *via* mass balance calculations. After all data sets had been analyzed, the structural results were compiled into a single text file by *gsas_prepare_cinema* and merged with sample parameters such as annealing temperature and time. A metallographic interpretation of the results is the subject of a future publication.

#### 3.1.1. Validation   

**Figure 2:**
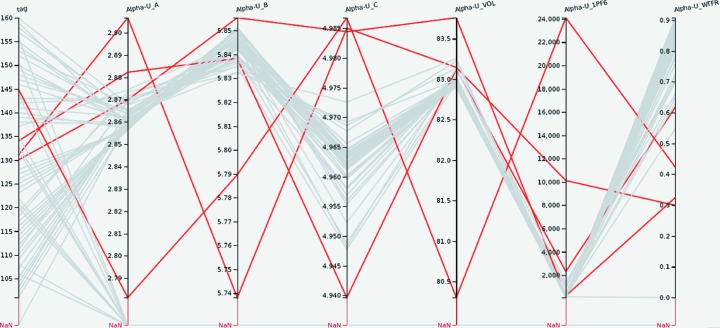
A parallel coordinates plot of sample identifier, lattice parameters 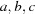, unit-cell volume, peak width parameter and weight fraction of α-uranium (axes from left to right). The red highlighted paths show outliers for the lattice parameters.

After development of the analysis script with a few data sets, all 59 data sets were analyzed in batch mode. To illustrate the ability of *Cinema:Debye-Scherrer* to identify potential problems in some data sets, Fig. 2 ▸ shows the lattice parameters, unit-cell volume, peak width parameter and weight fraction of α-U. While the majority of the analyses provided lattice parameters with a small variation, several analysis runs resulted in lattice parameters clearly outside the typical values from the majority of the 59 data sets. The paths of those runs are marked. It becomes immediately obvious that only two of the four outliers in the lattice parameters also result in distinct outliers for the unit-cell volume, *i.e.* in some cases the lattice parameter deviations cancel each other out and inspection of the unit-cell volume alone would not have revealed these outliers. Furthermore, in three of the four cases the peak width parameters are also above the typical values. In all cases, the weight fraction of α-U is low. These findings immediately caution the analyst on the results for these runs. The ability to select runs within a range on any of the axes allows inspection of the diffraction patterns produced by *GSAS* for these particular samples, including the difference curve. This may lead to insight into common problems with the Rietveld analysis of problematic analyses (see Fig. 1 ▸).

#### 3.1.2. Parameter relationships   

**Figure 3:**
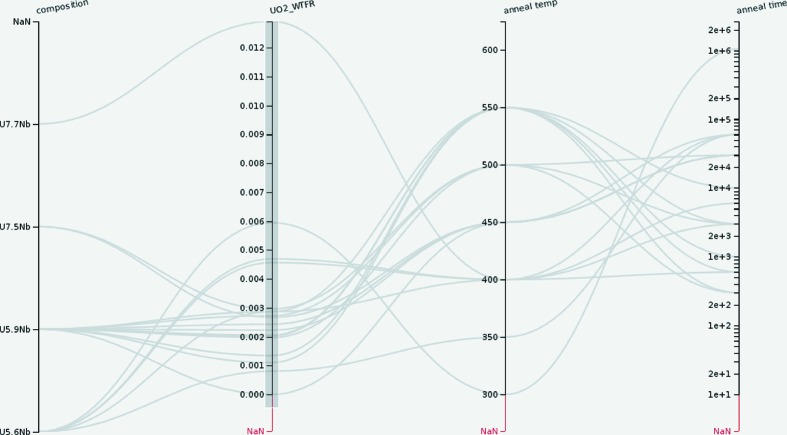
A parallel coordinates plot of annealing temperature, annealing time and the weight fraction of the UO_2_ phase. The bar on the UO2_WTFRAC (UO_2_ weight fraction) axis shows the selected paths with samples that have a non-zero weight fraction for the UO_2_ phase.

To illustrate the ability to gain scientific insight from a data set, Fig. 3 ▸ shows composition, annealing temperature and time together with the urania weight fraction. Selecting the entire range of weight fractions on the corresponding axis hides the paths of samples where no urania was found (indicated by ‘NaN’ on that axis). The parallel coordinates plot shows that oxidation predominantly occurred for medium annealing times and, except for three samples, only for the U–5.6Nb and U–5.9Nb samples. While this could indicate a higher corrosion resistance of U–7.5Nb and U–7.7Nb, in this case it is simply due to sample preparation.

**Figure 4:**
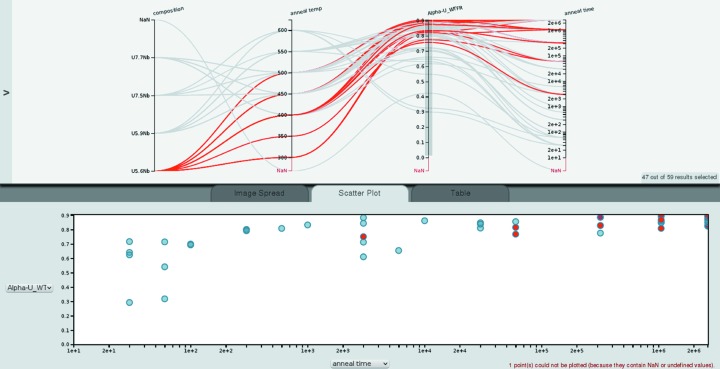
Full view of the *Cinema:Debye-Scherrer* interface with a scatter plot. The parallel coordinates plot shows composition, annealing temperature (in °C), α-U weight fraction and annealing time (in min).

**Figure 5:**
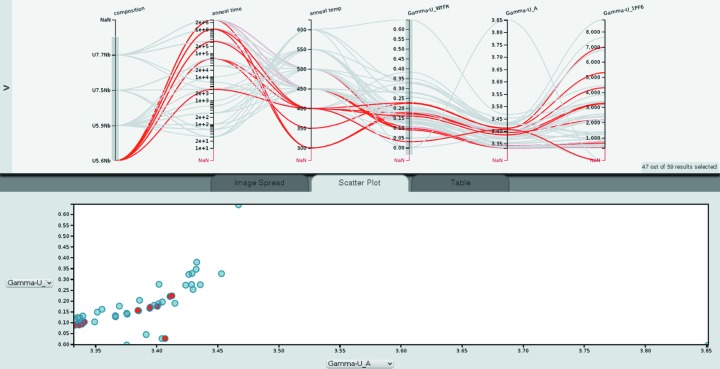
Parallel coordinates plot showing composition, annealing temperature (in °C), annealing time (in min), γ-U lattice parameter *a* (in Å) and weight fraction. The scatter plot shows γ-U weight fraction *versus* γ-U lattice parameter.

Fig. 4 ▸ shows composition, annealing temperature, annealing time and α-U weight fraction. The paths marked in red correspond to U–5.6Nb samples. The data points marked in red in the multi-axis plot are also marked in red in the scatter plot. As introduced in §2, the number of crossings in the parallel coordinates plot can be interpreted visually as a correlation, anti-correlation or absence of a correlation: Few crossings indicates a positive correlation since this means as one parameter increases, so does the other parameter. A strong negative correlation is represented by most or even all the paths crossing in a single point, while many crossings throughout the space between the axes indicates no correlation. Since the axes can be re-arranged by dragging the axis labels, the analyst can inspect correlations between two parameters by positioning the axes of two parameters next to each other. This allows us to inspect correlations between α-U weight fraction, annealing time and annealing temperature. The many crossings of paths between annealing temperature and α-U weight fraction indicate that there is no correlation between the two parameters. On the other hand, there are few paths crossing between the α-U weight fraction and the annealing time, indicating that increasing annealing time leads to increased α-U weight fraction. The scatter plot of all α-U weight fractions against annealing time confirms this trend. This behavior is typical of what is captured in typical time–temperature–transformation (TTT) diagrams. These diagrams typically show so-called ‘noses’ where, at certain temperatures in the intermediate temperature range, short times are sufficient to transform significant volume fractions of the material while above and below the ‘nose’ longer times are required. While the general trend that longer annealing times lead to larger volume fractions of a new phase, α-U in this case, holds for the U–Nb system characterized here, the TTT noses prevent a ‘perfect’ correlation with temperature. Similarly, the few crossings in Fig. 2 ▸ between the *c* lattice parameter and the unit-cell volume indicate the in this case obvious correlation between lattice parameter and unit-cell volume. The few crossings in Fig. 5 ▸ between the γ-U lattice parameter and the weight fraction of the same phase indicate a correlation between those parameters (see below). *Cinema:Debye-Scherrer* would allow ‘discovery’ of such systematics.

#### 3.1.3. Experiment design   

Both charts in Fig. 4 ▸ show that for the U–5.6Nb composition (marked in red) no samples with short annealing times were measured. This could indicate problems with the experiment design; however, in the present case these samples were not yet characterized.

Fig. 5 ▸ shows composition, annealing time and temperature, γ-U lattice parameter, and weight fraction. Almost no crossings of paths of the γ-U lattice parameter *a* and γ-U weight fraction are observed, indicating a correlation between the two parameters. The scatter plot of γ-U weight fraction *versus* γ-U lattice parameter shows a linear relationship for a large range of lattice parameters, which was first identified by Jackson (1970 ▸). However, our data show that for larger lattice parameters the relation might be nonlinear.

### 3.2. CsPbBr_3_   

The crystal structure of caesium plumbo tribromide, CsPbBr_3_, was investigated by neutron diffraction as a function of temperature using HIPPO (see above for a description and references). The material has a perovskite-type structure with orthorhombic crystal structure at room temperature and undergoes phase transformations to tetragonal and cubic crystal structures at 88 and 133°C, respectively (Møller, 1959 ▸; Hirotsu *et al.*, 1974 ▸; Stoumpos *et al.*, 2013 ▸). Neutron diffraction patterns were collected in 10°C steps from 35 to 175°C and in greater increments up to 400°C. Results of this study will be reported in a forthcoming paper.

#### 3.2.1. Validation   

**Figure 6:**
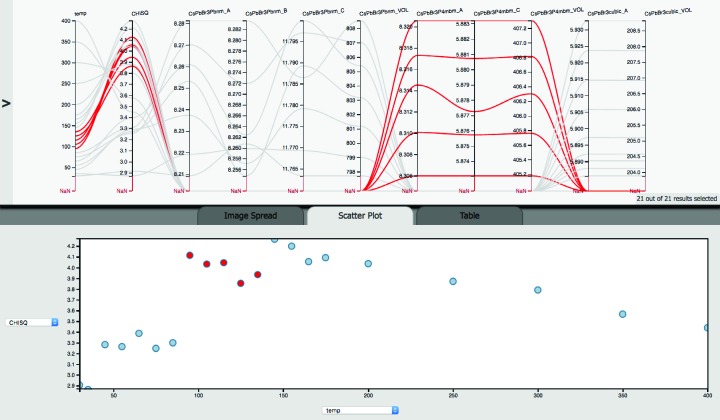
Multi-axis plot of temperature, reduced 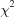, lattice parameters and unit-cell volumes of orthorhombic, tetragonal and cubic CsPbBr_3_. The scatter plot shows the reduced 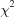 as a function of temperature.

Fig. 6 ▸ shows sample temperature, reduced 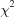, lattice parameters and unit-cell volumes of the three polymorphs. The paths between temperature and reduced 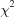 show that the runs in the temperature range of the ortho­rhombic phase have lower reduced 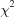 values, and therefore better fit quality, than the tetragonal phase, with the latter in turn resulting in worse agreement between experimental data and Rietveld fit than the cubic phase (except for the two highest temperatures). The scatter plot at the bottom of Fig. 6 ▸ confirms this. The crossover between the axes for the ortho­rhombic lattice parameters *a* and *b* indicates peculiarities in the thermal expansion behavior. The paths for the tetragonal phase, highlighted in red, show approximately constant increments in the unit-cell volume for the 10°C increments. However, the center path of the five paths shows a higher *a* lattice parameter with *c* lower than the remaining increments. This peculiarity would have gone undetected if only the reduced 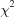, not different from the other 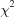 for this phase, or only the unit-cell volume had been considered. Alternatively, for experiment design or if detected by real-time data analysis and visualization, more data points could be collected in that temperature range to establish whether this observation is an artifact of the data analysis.

#### 3.2.2. Parameter relationships   

**Figure 7:**
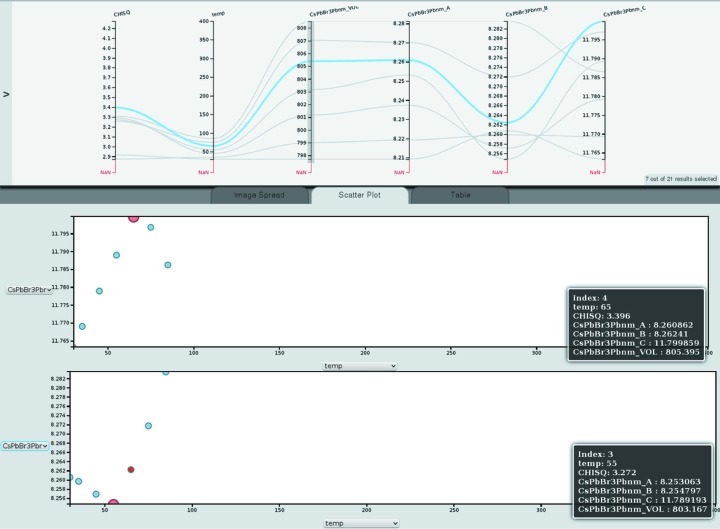
Multi-axis plot of reduced 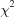, temperature, unit-cell volume and lattice parameters of the orthorhombic phase of CsPbBr_3_. The scatter plots show the *c* (middle) and *b* (bottom) lattice parameter as a function of temperature.

Fig. 7 ▸ shows the reduced 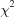, temperature, unit-cell volume and lattice parameters of the orthorhombic phase of CsPbBr_3_ only (by selecting the range on the unit-cell volume axis with values, excluding the runs for which this parameter is not available). The unit-cell volume increases with approximately constant increments when the temperature is increased. Since there are no paths crossing between unit-cell volume and lattice parameter *a*, the same is true for *a*. However, between lattice parameters *a* and *b* as well as between *b* and *c* crossing occurs, indicating deviations from that proportionality. The scatter plot of the lattice parameter *c* as a function of temperature shows that after an initial expansion along this direction, CsPbBr_3_ starts to contract at 65°C along this direction, leading to the crossings in the paths in the multi-axis plot.

#### 3.2.3. Experiment design   

As discussed above, *Cinema:Debye-Scherrer* allowed us to identify changes in the increments of *a* and *c* in the tetragonal phase while the unit-cell volume shows a constant increment with a constant temperature increment. If this discrepancy had been discovered during the experiment, this region could have been re-investigated. Furthermore, a reversal from expansion to contraction of the *c* axis was identified during heating together with a change from contraction to expansion along *b*. If this information had been available from real-time analysis during the experiment, this temperature region could have been re-investigated with smaller temperature increments.

## 4. Summary and conclusions   

The *Cinema:Debye-Scherrer* tool for multi-dimensional data visualization was described. Applying the tool to diffraction data sets from 59 U–Nb samples of different composition, annealing times and annealing temperatures and a study to investigate the crystal structure evolution of CsPbBr_3_ as a function of temperature illustrated how this tool provides an efficient overview of Rietveld analysis results. The required data are extracted from standard results files of the widely used *GSAS* Rietveld analysis software. Since *Cinema:Debye-Scherrer* is based on CSV files, similar files can be produced for other analysis packages. The tool allows us to efficiently identify outliers of automated Rietveld analysis runs and at the same time offers efficient access to the graphical output of diffraction data, Rietveld fit and difference curves for multi-histogram refinements. In the case of the U–Nb samples, the automated Rietveld analysis takes several minutes per data set owing to the complexity of the analysis, simultaneous refinement of multiple histograms and the multitude of parameters in the phases with low crystal symmetry present in the samples. The corresponding gsaslanguage command allows us to gather all required information after the automated analysis within seconds and *Cinema:Debye-Scherrer* displays results instantaneously. Visualization of the parameter space covered during an experiment (annealing times and temperatures in this example) identifies gaps in experimental coverage. The presence or absence of crossing points in the paths between two axes allows us to quickly identify correlations, which can then be further inspected using scatter plots. The latter serves as an example of how new scientific insight can be gained at an accelerated pace. All three aspects, (i) quality control of (automated) Rietveld analysis, (ii) identification of gaps in the experimental parameter space and (iii) accelerated scientific insight, are great assets in decision making during beam times when real-time data analysis is available. The tool has already proven useful for the management of large-scale sample sets and parametric studies at LANSCE. The visualization tool *Cinema:Debye-Scherrer* and the script for the extraction of *GSAS* Rietveld analysis results are available for download at the web sites provided in §2.1.4.

## References

[bb1] Arnold, O. *et al.* (2014). *Nucl. Instrum. Methods Phys. Res. A*, **764**, 156–166.

[bb2] Barr, G., Dong, W. & Gilmore, C. J. (2009). *J. Appl. Cryst.* **42**, 965–974.

[bb3] Bostock, M., Ogievetsky, V. & Heer, J. (2011). *IEEE Trans. Vis. Comput. Graph.* **17**, 2301–2309. 10.1109/TVCG.2011.185 22034350

[bb5] Hackenberg, R. E., Hemphill, G., Forsyth, R. T., Papin, P. A., Kelly, A. M., Tucker, T. J., Aikin, R. M. Jr, Alexander, D. J., Lopez, M. F. & Clarke, A. J. (2017). *Mater. Sci. Forum*, **879**, 665–670.

[bb6] Hackenberg, R. E., Volz, H. M., Papin, P. A., Kelly, A. M., Forsyth, R. T., Tucker, T. J. & Clarke, K. D. (2011). *Solid State Phenom.* **172–174**, 555–560.

[bb4] Hackenberg, R., Yablinsky, C., Llobet, A., Volz, H., Papin, P., Tucker, T., Clarke, K. & Emigh, M. (2015). *Proceedings of the International Conference on Solid–Solid Phase Transformations* (PTM-2015), edited by M. Militzer, G. Botton, L. Chen, J. Howe, C. Sinclair & H. Zurob, pp. 211–218. Warrendale: TMS.

[bb7] Hirotsu, S., Harada, J., Iizumi, M. & Gesi, K. (1974). *J. Phys. Soc. Jpn*, **37**, 1393–1398.

[bb8] Jackson, R. J. (1970). *Reversible Martensitic Transformations Between Transition Phases of Uranium-Base Niobium Alloys*. Technical Report RFP-1535 (23 December 1970). Rocky Flats Plant, Golden, CO, USA.

[bb9] Jackson, R. J. (1971). *Isothermal Transformations of Uranium-13 Atomic Percent Niobium*. Technical Report RFP-1609 (9 April 1971). Rocky Flats Plant, Golden, CO, USA.

[bb10] Larson, A. C. & Von Dreele, R. B. (2004). *General Structure Analysis System* (*GSAS*). Technical Report LAUR 86–748. Los Alamos National Laboratory, New Mexico, USA.

[bb11] Lisowski, P. W. & Schoenberg, K. F. (2006). *Nucl. Instrum. Methods Phys. Res. A*, **562**, 910–914.

[bb12] Losko, A. S., Vogel, S. C., Reiche, H. M. & Nakotte, H. (2014). *J. Appl. Cryst.* **47**, 2109–2112.

[bb13] Lutterotti, L. (2011). *Quantitative Rietveld Analysis in Batch Mode with Maud* (accessed February 2018), http://www.ing.unitn.it/~maud/tutorial/Maudbatch.pdf

[bb14] Lutterotti, L., Matthies, S., Wenk, H.-R., Schultz, A. S. & Richardson, J. W. Jr (1997). *J. Appl. Phys.* **81**, 594–600.

[bb15] Møller, C. K. (1959). *The Structure of Perovskite-Like Caesium Plumbo Trihalides*. Copenhagen: Munksgaard.

[bb16] Momma, K. & Izumi, F. (2011). *J. Appl. Cryst.* **44**, 1272–1276.

[bb17] O’Leary, P., Ahrens, J., Jourdain, S., Wittenburg, S., Rogers, D. H. & Petersen, M. (2016). *Parallel Comput.* **55**(C), 43–48.

[bb18] Rietveld, H. M. (1969). *J. Appl. Cryst.* **2**, 65–71.

[bb19] Stoumpos, C. C., Malliakas, C. D., Peters, J. A., Liu, Z., Sebastian, M., Im, J., Chasapis, T. C., Wibowo, A. C., Chung, D. Y., Freeman, A. J., Wessels, B. W. & Kanatzidis, M. G. (2013). *Cryst. Growth Des.* **13**, 2722–2727.

[bb20] Tian, P., Zhou, W., Liu, J., Shang, Y., Farrow, C. L., Juhás, P. & Billinge, S. J. L. (2013). *J. Appl. Cryst.* **46**, 255–258.

[bb21] Toby, B. H. & Von Dreele, R. B. (2013). *J. Appl. Cryst.* **46**, 544–549.

[bb22] Vogel, S. C. (2011). *J. Appl. Cryst.* **44**, 873–877.

[bb23] Wenk, H.-R., Lutterotti, L. & Vogel, S. (2003). *Nucl. Instrum. Methods Phys. Res. A*, **515**, 575–588.

[bb24] Wenk, H.-R., Lutterotti, L. & Vogel, S. (2010). *Powder Diffr.* **25**, 283–296.

[bb25] Young, R. A. (1993). *The Rietveld Method*. IUCr Monographs on Crystallography 5. Chester, Oxford: IUCr/Oxford University Press.

